# Designing synbiotics for improved human health

**DOI:** 10.1111/1751-7915.12885

**Published:** 2017-12-04

**Authors:** Sean M. Kearney, Sean M. Gibbons

**Affiliations:** ^1^ Department of Biological Engineering Massachusetts Institute of Technology Cambridge MA USA; ^2^ The Broad Institute of MIT and Harvard Cambridge MA USA; ^3^ Center for Microbiome Informatics and Therapeutics Cambridge MA USA

A synbiotic is the combination of a microorganism shown (or thought) to have some beneficial effect when consumed (i.e. a probiotic) and a compound that specifically favours its growth (i.e. a prebiotic), having a synergistic effect when paired together. Many probiotic supplements are currently marketed as synbiotics. These products typically contain a combination of *Bifidobacterium*,* Lactobacillus* or *Streptococcus* species, and a carbon substrate (e.g. lactose, lactulose or inulin) supporting growth of these organisms. Historical use of probiotics in foods and beverages and marketing towards digestive health has favoured food companies as producers of these products. The largest market share for probiotics comes from companies like Danone, Nestlé and General Mills, which are currently investing in R&D to expand their probiotic and prebiotic portfolios. Examples of growing interest in probiotics can be found among recent patents, containing claims of probiotics for reduction of belly fat (Grompone *et al*., 2014) or prebiotic fibre formulations for the treatment of inflammatory bowel disease (Boileau *et al*., 2015). In coming years, focus on the microbiome may shift the market share of probiotics towards pharmaceutical companies, which have infrastructure and revenue models to accommodate clinical trials. Indeed, both existing pharmaceutical companies (e.g. Johnson and Johnson, Merck, Pfizer and Novartis) and recent start‐ups (e.g. Vedanta, Eligo Biosciences, Finch Therapeutics) have begun to target probiotics from the human microbiota for treatment of a broad range of diseases.

Currently available probiotics sample a limited diversity of bacteria that does not include most dominant gut microorganisms positively associated with host health. The use of these established probiotics stems from their historical association with improved digestive health. Today, probiotic, prebiotic and synbiotic products are marketed towards use in gynaecology, urology, anti‐ageing, gastroenterology, immunology, cardiology, skin care, dietetics and oral care. The broad applications of this limited clique of organisms suggest that there is a need for more extensive clinical and epidemiological evaluation of probiotics and their efficacies in the treatment of a variety of conditions. In general, for probiotics to be marketed as pharmaceutical products, the burden of proof for efficacy will be much greater than for similar formulations marketed as functional food products, similar to vitamins or other over‐the‐counter supplements.

For well‐studied probiotic species, we have some understanding of the mechanisms by which they impact host health. Some *Lactobacillus* species, in particular, are thought to deplete systemic pro‐inflammatory Th17 immune cells through the production of tryptophan metabolites, which activate the aryl hydrocarbon receptor (AHR; Zelante *et al*., 2013). Many of their other downstream effects on host health have yet to be elucidated, but there are indications that certain *Lactobacillus* species may affect brain (Bravo *et al*., 2011), reproductive (Linhares *et al*., 2011) and epithelial barrier function (Levkovich *et al*., 2013). Ongoing research on the diverse and understudied members of the human gut microbiota likely will propel pharmaceutical investment in clinically relevant probiotics and synbiotics. By contrast, food and probiotics companies will likely invest in formulations that incorporate well‐studied, previously identified probiotics to promote their non‐clinical use in different settings.

Recent studies analysing the effects of introduced bacteria on the host suggest that several commensals have generic and redundant effects on immunity (Geva‐Zatorsky *et al*., 2017). However, these effects may change in the presence of an intact microbial community and likely interact with diet and host health (Maldonado‐Gómez *et al*., 2016). Some of these interactions may be desirable: e.g., some probiotics show competitive exclusion of pathogenic organisms (Caballero *et al*., 2017), support the growth of native bacteria helpful for host health (Belzer *et al*., 2017; Caballero *et al*., 2017) and provision nutrients from the diet otherwise inaccessible to the host (Marcobal and Sonnenburg, 2012). The design and use of probiotics and prebiotics should reflect these considerations. Below, we outline such considerations for the design of effective synbiotics and highlight opportunities for new economic growth in this field.

## Design criteria for synbiotics

### Probiotics promote specific, desired effects on the host

Many probiotic organisms currently on the market claim to support digestive health and often are used for indications such as diarrhoea or constipation. The market for probiotics is likely to increase and marketing of these organisms may extend beyond digestive diseases. Given the pace of research on the health potential of the human microbiome, next‐generation probiotics should have some demonstrated health effect, based on either clinical trials in humans or on animal studies. However, inferred effects may be sufficient to justify probiotic use in the absence of negative health effects. The start‐up company AOBiome uses this strategy to market ammonia‐oxidizing bacteria that putatively reduce odours from sweat when applied to skin (Whitlock, 2010), although there is little evidence to support this claim. This topically applied probiotic provides a window into the potential uses of probiotic organisms that extend beyond historical applications in digestive health.

### Probiotics are safe

Most probiotics used in the USA are generally regarded as safe (GRAS). This designation is important for probiotics, but is generally restricted in scope to those organisms that are involved in the fermentation of food products such as yogurt or cheese. GRAS status for other probiotics comes from a consensus of medical and scientific observations. For manufacturing purposes, organisms with GRAS status are easier to bring to market, and diminish concerns about potential health risks associated with consumption of a particular probiotic. However, many potentially therapeutic microbes [e.g. Clostridia species that reside in the human gut (Atarashi *et al*., 2011, 2013; Stefka *et al*., 2014; Yano *et al*., 2015; Kim *et al*., 2017)] currently lack GRAS status and are not sold as probiotics. It will be important for researchers and clinicians to work closely with government regulatory agencies to develop guidelines for designating human gut commensals as GRAS organisms. The rapid expansion in the use of faecal microbiota transplant (FMT) for the treatment of recurrent *C. difficile* infection was enabled in large part by a close collaboration between the non‐profit organization OpenBiome and clinical collaborators across the USA. Similar partnerships between clinical collaborators and academic partners can help accelerate the pace of research into the safety and efficacy of probiotic supplements in a range of applications.

### Probiotics are impermanent

It is important to specify appropriate indications for each probiotic because the health status of patients will in some cases alter the pathogenic potential of consumed probiotics. For this reason, probiotic organisms should be transient in their hosts to avoid potential long‐term negative health consequences. This criterion also satisfies a commercial incentive in designing probiotics that require repeated introduction to confer their health effects. Thus, probiotics should be temporary residents of their host, washing out of the system several days or weeks after cessation of their consumption. Most currently marketed probiotics fall into this category, such as *Lactobacilli*,* Bifidobacterium* and *Streptococcus*. Alternatively, if probiotics were to colonize a host for longer periods of time, then this colonization should be reversible. For example, if a probiotic requires a particular compound in order to remain in the system, then modulating this substrate as a prebiotic could control the probiotic's presence or absence. To track the stability of an introduced probiotic over time, companies could offer microbiome‐sequencing services, similar to those provided by the American Gut Project or uBiome, for users of their product in exchange for a nominal fee. These data would contribute significantly to understanding the factors that influence retention or loss of these organisms in the microbiome.

### Prebiotics selectively promote the growth of intended bacteria

Prebiotics can encompass a broad array of compounds and growth factors, which, when combined with a probiotic, promote its growth in the system. Most prebiotic compounds currently in use support the growth of many different bacteria indigenous to the host, so there must be some indication that a particular prebiotic specifically supports the growth of the introduced organism for the pair to be referred to as a synbiotic. One approach to the design of synbiotics is through *in vivo* enrichment with a prebiotic of interest and subsequent isolation of organisms whose abundance increases with the introduced prebiotic (Krumbeck *et al*., 2015). This approach ensures that even in a competitive background, the isolated organisms will be the most likely to use the prebiotic compound of interest. Another approach is to start with a prebiotic compound that can be used by a specific probiotic, but this compound is inaccessible to the endogenous community (e.g. a genetically engineered probiotic that can break down some exotic substrate). In any case, prebiotics in theory can be used in the absence of probiotics, with the intention of enriching for commensals already living within a person. For example, General Mills is working on a prebiotic formulation that enriches for specific commensals for the treatment of inflammatory bowel disease (Boileau *et al*., 2015). Gathering these data in an academic context can be challenging given limited study populations, but academic and industrial or clinical partnerships can greatly extend our understanding of how specific prebiotic or dietary compounds interact with individual members of the gut microbiota by recruiting large patient/consumer cohorts.

### Prebiotics can be present in the host or introduced with the probiotic

Prebiotics should support the growth of introduced probiotics, but need not be directly supplied with the probiotic. For example, *Oxalobacter formigenes*, an oxalate‐degrading bacterium resident to the gut microbiota, depends on the presence of oxalate, and removal of oxalate from the diet should discontinue the growth of this microorganism. The presence of both oxalate and *Oxalobacter formigenes* forms a synbiotic, allowing it to confer health benefits to the host through the degradation of oxalate, the accumulation of which can cause kidney stones (Kaufman *et al*., 2008). In this vein, well‐designed probiotics can make use of nutrients already present in the host as prebiotics, creating a synbiotic without directly supplying the prebiotic. Similarly, others have shown that the persistence of an introduced *Bifidobacterium* probiotic has two requirements: (i) presence of an endogenous nutrient source and (ii) absence of competition for that nutrient source by other commensals (Maldonado‐Gómez *et al*., 2016). Thus, design of prebiotics can make use of knowledge about either abundant energy sources not exploited by endogenous commensals or energy sources initially absent from the host and favouring the growth of the introduced probiotic over endogenous commensals.

### Prebiotics should not cause harm

Again using oxalate as an example, oxalate itself could be harmful in the absence of an organism like *Oxalobacter formigenes* that can degrade it. Common prebiotics, like inulin, can lead to excessive gas production, which can cause discomfort or pain (Slavin, 2013). In a synbiotic combination, prebiotics should be selected to increase the abundance of the target probiotic organism to promote growth without inducing rapid fermentation and gas production. Selection of more complex fermentable fibres to enrich for target organisms may reduce these concerns.

### Prebiotics do not have to be carbon substrates

The AOBiome example of using ammonia‐oxidizing bacteria highlights other potential elements in the system, such as nitrogen, phosphorous or iron, which may allow for design of targeted prebiotics that promote respiration over fermentation. *Bilophila wadsworthia*, for example, uses the sulfite present in taurine for dissimilatory sulfite reduction (Devkota *et al*., 2012). In fact, strategies that promote alternative modes of respiration are of great relevance in agriculture, where methane production by cows is driven by methanogenic archaea. Inhibition of methanogenesis shifts respiration to acetogenesis, and actually promotes energy harvest by cows while decreasing the environmental burden of methane production (Hristov *et al*., 2015). Many agricultural companies are starting to invest in prebiotic and probiotic supplements for a range of applications, both to reduce reliance on antibiotics and to satisfy consumers’ growing concerns over animal welfare and environmental impacts of industrial agriculture. In a new partnership between Bayer and Ginkgo Bioworks, engineers are hoping to use nitrogen fixation capacity of legume‐associated bacteria to reduce reliance on chemical fertilizers, using abundant atmospheric nitrogen as a route to improving plant growth.

### Synbiotics should have a greater effect than the prebiotic or probiotic alone

The word synbiotic implies a synergistic effect from the probiotic and prebiotic. The design of a synbiotic, then, requires only that the probiotic exerts a greater effect in the presence of a prebiotic than in its absence, and vice versa. Unfortunately, few experiments address this consideration: prebiotics are not routinely combined with a corresponding probiotic to show an increased benefit to the host than in the absence of either component, as was the case in a recent synbiotic trial to prevent sepsis among infants in rural India (Panigrahi *et al*., 2017). Future experimental work on the effects of synbiotics should assess not only the effect of the prebiotic on the probiotic, but the change in effect on the host in combination versus in isolation. Justification for the use of synbiotics ultimately requires such proof, and obtaining this evidence will entail major investments from the key academic, industrial and clinical players in this field.

## Outlook

Knowledge about the human microbiota will improve our ability to design synbiotics for a variety of applications. Next‐generation probiotics should have well‐demonstrated effects on the host, be safe to use and be reversible colonizers. Synbiotic combinations will make use of prebiotics that selectively promote the growth of introduced probiotics, with or without being directly supplied with the probiotic. These synbiotics offer a promising solution to public health problems ranging from digestive disease to skin health. Continual monitoring of probiotics and their impacts on health will prove increasingly important, as it is difficult to predict long‐term effects of introduced microorganisms. However, targeted use of probiotics for specific indications and selection of transient organisms should ameliorate such concerns. Low‐cost synbiotics have the potential to address a number of public health concerns while expanding our understanding of gut ecology. Finally, the commercial and clinical use of synbiotics will likely create many economic opportunities and contribute to greater public awareness of the beneficial microorganisms living in and on us.

## Conflict of interest

None declared.

## References

[mbt212885-bib-0001] Atarashi, K. , Tanoue, T. , Shima, T. , Imaoka, A. , Kuwahara, T. , Momose, Y. , *et al* (2011) Induction of colonic regulatory T cells by indigenous clostridium species. Science 331: 337–341. 21205640 10.1126/science.1198469 PMC3969237

[mbt212885-bib-0002] Atarashi, K. , Tanoue, T. , Oshima, K. , Suda, W. , Nagano, Y. , Nishikawa, H. , *et al* (2013) Treg induction by a rationally selected mixture of clostridia strains from the human microbiota. Nature 500: 232–236. 23842501 10.1038/nature12331

[mbt212885-bib-0003] Belzer, C. , Chia, L.W. , Aalvink, S. , Chamlagain, B. , Piironen, V. , Knol, J. and de Vos, W.M. (2017) Microbial metabolic networks at the mucus layer lead to diet‐independent butyrate and vitamin B(12) production by intestinal symbionts. mBio 8, e00770–17. 28928206 10.1128/mBio.00770-17 PMC5605934

[mbt212885-bib-0004] Boileau, T.W. , Brulc, J. and Menon, R.S. (2015) Methods and compositions for modulating gastrointestinal bacteria to promote health.

[mbt212885-bib-0005] Bravo, J.A. , Forsythe, P. , Chew, M.V. , Escaravage, E. , Savignac, H.M. , Dinan, T.G. , *et al* (2011) Ingestion of Lactobacillus strain regulates emotional behavior and central GABA receptor expression in a mouse via the vagus nerve. Proc Natl Acad Sci USA 108: 16050–16055. 21876150 10.1073/pnas.1102999108 PMC3179073

[mbt212885-bib-0006] Caballero, S. , Kim, S. , Carter, R.A. , Leiner, I.M. , Sušac, B. , Miller, L. , *et al* (2017) Cooperating commensals restore colonization resistance to vancomycin‐resistant enterococcus faecium. Cell Host Microbe 21: 592–602. 28494240 10.1016/j.chom.2017.04.002 PMC5494988

[mbt212885-bib-0007] Devkota, S. , Wang, Y. , Musch, M.W. , Leone, V. , Fehlner‐Peach, H. , Nadimpalli, A. , *et al* (2012) Dietary‐fat‐induced taurocholic acid promotes pathobiont expansion and colitis in Il10−/− mice. Nature 487: 104–108. 22722865 10.1038/nature11225 PMC3393783

[mbt212885-bib-0008] Geva‐Zatorsky, N. , Sefik, E. , Kua, L. , Pasman, L. , Tan, T.G. , Ortiz‐Lopez, A. , *et al* (2017) Mining the human gut microbiota for immunomodulatory organisms. Cell 168: 928–943. 28215708 10.1016/j.cell.2017.01.022 PMC7774263

[mbt212885-bib-0009] Grompone, G. , Ramon, V.D. , Martorell, G.P. , Genovés, M.S. , Ortiz, S.P. , Llopis, P.S. and Gonzalez, M.N. (2014) Lactobacillus rhamnosus strain for reducing body fat accumulation.

[mbt212885-bib-0010] Hristov, A.N. , Oh, J. , Giallongo, F. , Frederick, T.W. , Harper, M.T. , Weeks, H.L. , *et al* (2015) An inhibitor persistently decreased enteric methane emission from dairy cows with no negative effect on milk production. Proc Natl Acad Sci 112: 10663–10668. 26229078 10.1073/pnas.1504124112 PMC4553761

[mbt212885-bib-0011] Kaufman, D.W. , Kelly, J.P. , Curhan, G.C. , Anderson, T.E. , Dretler, S.P. , Preminger, G.M. , and Cave, D.R. (2008) Oxalobacter formigenes may reduce the risk of calcium oxalate kidney stones. J Am Soc Nephrol 19: 1197–1203. 18322162 10.1681/ASN.2007101058 PMC2396938

[mbt212885-bib-0012] Kim, Y.‐G. , Sakamoto, K. , Seo, S.‐U. , Pickard, J.M. , Gillilland, M.G. , Pudlo, N.A. , *et al* (2017) Neonatal acquisition of *Clostridia* species protects against colonization by bacterial pathogens. Science 356: 315. 28428425 10.1126/science.aag2029 PMC6082366

[mbt212885-bib-0013] Krumbeck, J.A. , Maldonado‐Gomez, M.X. , Martínez, I. , Frese, S.A. , Burkey, T.E. , Rasineni, K. , *et al* (2015) In vivo selection to identify bacterial strains with enhanced ecological performance in synbiotic applications. Appl Environ Microbiol 81: 2455–2465. 25616794 10.1128/AEM.03903-14 PMC4357922

[mbt212885-bib-0014] Levkovich, T. , Poutahidis, T. , Smillie, C. , Varian, B.J. , Ibrahim, Y.M. , Lakritz, J.R. , *et al* (2013) Probiotic bacteria induce a “glow of health”. PLoS ONE 8: e53867. 23342023 10.1371/journal.pone.0053867 PMC3547054

[mbt212885-bib-0015] Linhares, I.M. , Summers, P.R. , Larsen, B. , Giraldo, P.C. and Witkin, S.S. (2011) Contemporary perspectives on vaginal pH and lactobacilli. Am J Obstet Gynecol 204, 120. e1–120.e5. 20832044 10.1016/j.ajog.2010.07.010

[mbt212885-bib-0016] Maldonado‐Gómez, M.X. , Martínez, I. , Bottacini, F. , O'Callaghan, A. , Ventura, M. , van Sinderen, D. , *et al* (2016) Stable engraftment of bifidobacterium longum AH1206 in the human gut depends on individualized features of the resident microbiome. Cell Host Microbe 20: 515–526. 27693307 10.1016/j.chom.2016.09.001

[mbt212885-bib-0017] Marcobal, A. , and Sonnenburg, J.L. (2012) Human milk oligosaccharide consumption by intestinal microbiota. Clin Microbiol Infect 18: 12–15. 22647041 10.1111/j.1469-0691.2012.03863.x PMC3671919

[mbt212885-bib-0018] Panigrahi, P. , Parida, S. , Nanda, N.C. , Satpathy, R. , Pradhan, L. , Chandel, D.S. , *et al* (2017) A randomized synbiotic trial to prevent sepsis among infants in rural India. Nature 548: 407–412. 28813414 10.1038/nature23480

[mbt212885-bib-0019] Slavin, J. (2013) Fiber and prebiotics: Mechanisms and health benefits. Nutrients 5: 1417–1435. 23609775 10.3390/nu5041417 PMC3705355

[mbt212885-bib-0020] Stefka, A.T. , Feehley, T. , Tripathi, P. , Qiu, J. , McCoy, K. , Mazmanian, S.K. , *et al* (2014) Commensal bacteria protect against food allergen sensitization. Proc Natl Acad Sci 111: 13145–13150. 25157157 10.1073/pnas.1412008111 PMC4246970

[mbt212885-bib-0021] Whitlock, D.R. (2010) Compositions including ammonia oxidizing bacteria to increase production of nitric oxide and nitric oxide precursors and methods of using same.

[mbt212885-bib-0022] Yano, J.M. , Yu, K. , Donaldson, G.P. , Shastri, G.G. , Ann, P. , Ma, L. , *et al* (2015) Indigenous bacteria from the gut microbiota regulate host serotonin biosynthesis. Cell 161: 264–276. 25860609 10.1016/j.cell.2015.02.047 PMC4393509

[mbt212885-bib-0023] Zelante, T. , Iannitti, R.G. , Cunha, C. , De Luca, A. , Giovannini, G. , Pieraccini, G. , *et al* (2013) Tryptophan catabolites from microbiota engage aryl hydrocarbon receptor and balance mucosal reactivity via interleukin‐22. Immunity 39: 372–385. 23973224 10.1016/j.immuni.2013.08.003

